# Gating of homeostatic regulation of intrinsic excitability produces cryptic long-term storage of prior perturbations

**DOI:** 10.1073/pnas.2222016120

**Published:** 2023-06-20

**Authors:** Leandro M. Alonso, Mara C. P. Rue, Eve Marder

**Affiliations:** ^a^Volen Center and Biology Department, Brandeis University, Waltham, MA 02454

**Keywords:** ion channels, negative feedback, neuronal oscillators, neuronal stability

## Abstract

Neurons and neuronal circuits must maintain their function throughout the life of the organism despite changing environments. Previous theoretical and experimental work suggests that neurons monitor their activity using intracellular calcium concentrations to regulate their intrinsic excitability. Models with multiple sensors can distinguish among different patterns of activity, but previous work using models with multiple sensors produced instabilities that lead the models’ conductances to oscillate and then to grow without bound and diverge. We now introduce a nonlinear degradation term that explicitly prevents the maximal conductances to grow beyond a bound. We combine the sensors’ signals into a master feedback signal that can be used to modulate the timescale of conductance evolution. Effectively, this means that the negative feedback can be gated on and off according to how far the neuron is from its target. The modified model recovers from multiple perturbations. Interestingly, depolarizing the models to the same membrane potential with current injection or with simulated high extracellular *K*^+^ produces different changes in conductances, arguing that caution must be used in interpreting manipulations that serve as a proxy for increased neuronal activity. Finally, these models accrue traces of prior perturbations that are not visible in their control activity after perturbation but that shape their responses to subsequent perturbations. These cryptic or hidden changes may provide insight into disorders such as posttraumatic stress disorder that only become visible in response to specific perturbations.

## Significance

Neurons in long-lived animals must replace their ion channels and other membrane proteins many times during their life time. This updated model of homeostatic regulation of intrinsic activity recovers from many perturbations, is gated on and off according to the extent of deviation from desired activity levels, and can provide insight into hidden or cryptic changes that may be revealed by specific sets of environmental changes.

Neurons in healthy human brains routinely live for decades, but the proteins that determine and control their intrinsic excitability turn over in the membrane in hours, days, or weeks. It has long been recognized that this poses a challenge for long-lived excitable cells, if they are to maintain their characteristic intrinsic properties over years, as channel proteins are replaced. In response to this conundrum, a number of models of activity-dependent regulation of channel density, termed intrinsic homeostatic regulation, have been developed (1–13). A number of experimental studies are loosely consistent with the principles of these models (14–21).

While existing models have different implementations and features, they share the concept that the neuron can sense its own activity patterns with intracellular *Ca*^2+^ concentrations and that changes in *Ca*^2+^ concentrations are used to alter the conductance densities of the ion channels expressed. In all of these models, the implicit assumption is that there is a target activity pattern and that changes in conductance densities are part of the control system to return the neuron to its target activity. Despite differences in implementation, these models all were designed to regulate a neuron to specific patterns of activity, such as firing action potentials or bursting at a specific average rate. Given a specific target, these models will self-assemble to a desired activity pattern (7, 11). Moreover, because the homeostatic feedback is assumed to be slow relative to the fast dynamics of the neuron’s activity, a neuron’s activity will recover from some of those perturbations.

There are multiple sets of maximal conductances that are consistent with a given activity pattern (22–29). Consequently, it is not surprising that homeostatic models do not necessarily return to the same starting point in conductance or stay there. Instead, they can achieve their target activity patterns with somewhat different sets of maximal conductances (4, 7, 11).

In this study, we illustrate that repeated patterns of exposure to a strong perturbation can lead to experience-dependent changes in conductance densities that can persist for a long-time. These changes can be cryptic, indeed invisible, unless the neuron is perturbed. We suggest that this model provides a heuristic way of understanding long-term changes in neuronal excitability that could be part of the kinds of changes in activity such as those in posttraumatic stress disorder, seizure disorders, or other states in which normal activity patterns are maintained under control conditions, but in which the system behaves abnormally only in response to a strong challenge.

## Results

### Review and Limitations of Liu et al. Model of Activity-Dependent Homeostasis.

In this work, we introduce a modification of the model in ref. 7. The model uses calcium currents to modify its conductance densities so that it will achieve a prescribed target activity. The model employs three sensors that monitor the total calcium current over different timescales and are named accordingly as fast (F), slow (S), and dc (D). The activities of the sensors are used to drive changes in the maximal conductances using the following equation:

[1] $$\begin{array}{c} \tau_{G}\frac{d\bar{g_{i}}}{\mathrm{dt}}=\left[A_{i}\left(\overset{¯}{F}-F\right)+B_{i}\left(\overset{¯}{S}-S\right)+C_{i}\left(\overset{¯}{D}-D\right)\right]\bar{g_{i}}. \end{array}$$

Here, $\overset{¯}{F}$, $\overset{¯}{S}$, and $\overset{¯}{D}$ are sensor targets for the activities of the sensors, and *τ*_*G*_ is the timescale of conductance evolution. Different choices of the sensor targets result in different patterns of activity ranging from various periodic bursting patterns to tonic spiking. The coefficients *A*_*i*_, *B*_*i*_, and *C*_*i*_ determine what the model will do with each conductance when the average activity of the corresponding sensor is off target. The coefficients used in ref. 7 are reproduced in Table 1.

**Table 1.** Control coefficients used in ref. 7

	Na	CaS	CaT	Kd	KCa	A	H
A	1	0	0	1	0	0	0
B	0	1	1	−1	−1	−1	1
C	0	0	0	0	−1	−1	1

The control scheme in Table 1 can be represented by a matrix **L**, and the distance of each sensor to its target can be represented by a vector $\delta =[(\overset{¯}{F}-F),(\overset{¯}{S}-S),(\overset{¯}{D}-D)]$. Using this notation, we can rewrite **1** as

[2] $$\begin{array}{c} \tau_{G}\frac{d\bar{g_{i}}}{\mathrm{dt}}=\left(\sum_{k}^{3}L_{\mathrm{ki}}\delta_{k}\right)\bar{g_{i}}. \end{array}$$

This model is different from most neuronal models because its conductance densities—which are typically treated as fixed parameters—correspond to state variables, and therefore, they change over time. The model in ref. 7 exhibits several remarkable properties. When started from small random conductance values, it can self-assemble into one of several possible activity patterns. For simplicity, in this work, we focused on periodic bursting patterns of activity. Different initial conditions (ie, initial conductances) result in similar bursting patterns with different conductance values. Importantly, when challenged by perturbations that disrupt the activity, in many cases, the model can regulate its conductances to recover its target activity. The activity-dependent homeostatic model in ref. 7 is consistent with individual variability—small variations in activity and conductance densities across individuals—as well as how neurons may recover from perturbations. Nonetheless, this model, as originally instantiated, becomes unstable, as discussed below.

We revisited some of the results in ref. 7. We searched for values of the sensors’ targets such that the model produces a periodic bursting pattern. We explored the dynamics of the models by repeating the simulations using a wide range of timescales ranging from 1 s to 20 min. Increasing *τ*_*G*_ results in equivalent but slower dynamics. For this reason, throughout this work, we refer to time both in conventional units such as time and seconds as well as in units of the relevant timescale in the model *τ*_*G*_. If we wanted to reproduce the experimental timescales of homeostatic processes in biological neurons, we would choose a much slower timescale *τ*_*G*_, on the order of hours. A faster timescale is useful to fully explore the models’ behaviors at a reasonable computational cost. Because we want to explore the long-term behavior and limits of these rules, in this work, we set the timescale of conductance evolution to *τ*_*G*_ = 2 s.

**Fig. 1.**
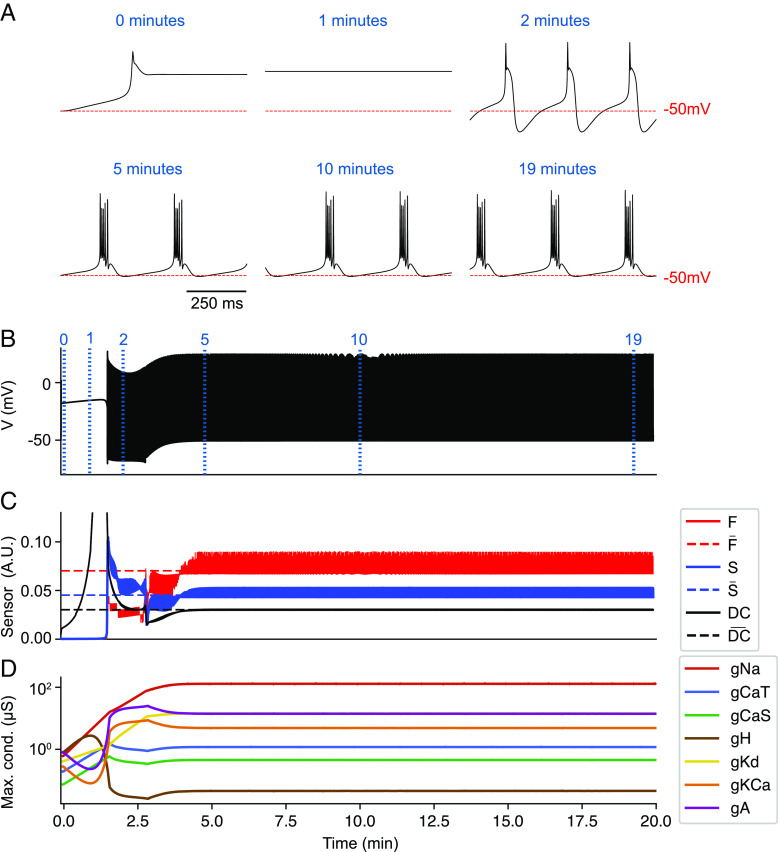
Liu model. The model is started from small random initial conditions and it self-assembles into its target pattern (periodic bursting). After achieving the target pattern, the conductances remain approximately constant for the rest of the simulation. (*A*) Membrane potential at different times in the simulation. (*B*) Membrane potential for the full simulation. (*C*) Sensors’ activities averaged over time (solid lines) and sensors’ targets (dashed lines). (*D*) Conductances over time on the logarithmic scale.

We started Liu’s model [**2**] from small random initial conductances and let it evolve for 1 h. Fig. 1 shows one simulation. The *Top* panels show traces at different points in time, indicated by the blue vertical lines and labels, and the full membrane potential envelope is displayed in Fig. 1*B*. Fig. 1*C* shows the time-averaged activity of the sensors (averaged over 2 s), and the dashed lines indicate the sensors’ targets. The *Bottom* panel (Fig. 1*D*) shows how the conductances change over time (on a logarithmic scale). Initially, the model becomes depolarized and quiescent (0 min). The sensors’ activities are off-target, and this in turn drives changes in the conductances. Two minutes later, the activity corresponds to a single-spike bursting mode. The model reaches its target activity at minute 5 and remains there for the rest of the simulation. The values of the conductances remain approximately constant, and in some cases, they display small oscillations because the sensors’ activities are also oscillatory.

Apparent convergence as shown in Fig. 1 can occur in this model when the sensors’ activities are on average close to the targets; then, ∥⟨***δ***⟩∥ ≈ 0 implies $\|\langle \overset{¯}{g}\rangle \|\approx 0$. However, it can be the case that the three sensors cannot be simultaneously satisfied, and this gives rise to run-away conductance evolution (11). This blow-up in conductance values and consequent instability was reported in the original study (7) as they found that some initial conditions lead to such behavior. Convergence in the sense that $\|\langle \overset{¯}{g}\rangle \|\approx 0$ is not guaranteed (and of course $\overset{¯}{g}$ cannot be zero; it can only approach 0 on average), and thus, some drifting of the conductance values is expected. Because the model is degenerate (many values of the conductances result in similar activities), there are regions in conductance space *G* such that a small change in conductances $d\overset{¯}{g}$ results in similar activity. In these cases, the direction $d\overset{¯}{g}$ has neutral stability: Changes along this direction will accumulate and result in a net drift of some conductance densities.

**Fig. 2.**
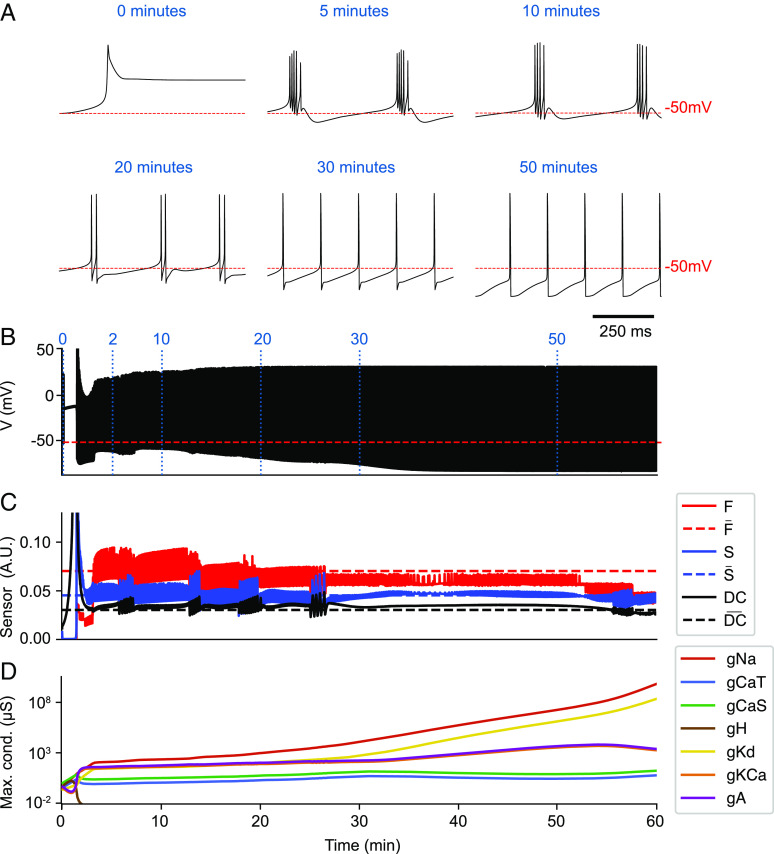
Instabilities result in divergence of maximal conductances. The model achieves the target bursting pattern, but conductances continue to change and diverge exponentially. (*A*) Membrane potential at different times in the simulation. (*B*) Membrane potential for the full simulation. (*C*) Sensors’ activities averaged over time (solid lines) and sensors’ targets (dashed lines). (*D*) Conductances over time in the logarithmic scale.

This can be seen in Fig. 2 where the exact same model as in Fig. 1 was started from different small random initial conductances. At first, the activity looked very similar to the previous case. By minute 5, the activity appeared to be on target, but the conductances never stopped evolving. Instead, by minutes 20 and 30, the waveform changed and spikes became more prominent. By minute 50, the cell was spiking tonically, some of the conductances grew by seven orders of magnitude, and diverged afterward. The reason the conductances kept evolving after minute 5 is that the waveforms at minutes 20, 30, and 50 correspond to calcium activities that activate the sensors in a similar way. Notice that the sensors’ activities in Fig. 2*C* at minute 50 are almost on target, but the cell is not bursting.

**Fig. 3.**
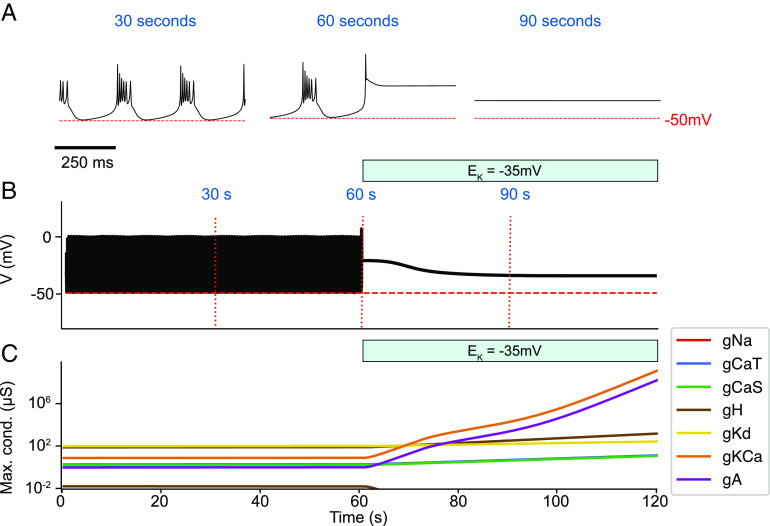
Failure to recover drives conductances to infinity. If the model cannot recover from a particular perturbation, it will continue to drive changes in its conductances. Since recovery is not possible for this particular perturbation, the homeostatic mechanism will not stop, and the conductances diverge exponentially. (*A*) Membrane potential at different times in the simulation. (*B*) Membrane potential for the full simulation. (*C*) Conductances over time in the logarithmic scale.

In Eq. **2**, there is nothing that prevents conductances from growing indefinitely. This results in an additional limitation to the model in ref. 7. If the model is subject to a perturbation that disrupts the activity and for which recovery is not possible, the homeostatic mechanism will keep changing the conductance densities, again resulting in diverging values for the conductances (Fig. 3*A*). The model was initially in a periodic bursting regime. A perturbation corresponding to changing the reversal potential of the potassium currents from *E*_*K*_ = −80 mV (control) to *E*_*K*_ = −35 mV was applied. The value of *E*_*K*_ during the perturbations was chosen so that the cell became quiescent (depolarization block) and could not recover. Fig. 3*A* shows how the conductance densities increased exponentially during the perturbation.

In summary, Liu’s model [**2**] has three main limitations.

- It sometimes blows up, and it is therefore unclear whether the models will really recover from a given perturbation since they can partially recover and then blow up (Fig. 2).
- If a perturbation is such that there is no possible recovery, the sensors will be far from their targets, and this can result in unbounded growth of the conductances (Fig. 3).
- The three sensors are not completely sufficient to fully characterize the waveforms. This translates into both false positives and false negatives: There are activity patterns that are not periodic bursting that activate the three sensors (Fig. 2*A*, minute [40]), and there are periodic bursting patterns that do not activate the sensors (not shown). One solution for this problem would be to try and find better sets of sensors and/or add more sensors. Despite this, the three sensors in Liu’s model work reasonably well, and we will not address this limitation.

Of course, three sensors might not be enough to characterize the membrane potential waveform or activity. This limitation can be substantially worse with only one sensor (6), which is what originally motivated Liu et al. (7) to include multiple sensors. In their work, Liu et al. (7) show how three different patterns of activity—tonic spiking, periodic bursting and single spike bursting—all correspond to the same average intracellular calcium. Importantly, this implies that models that rely on a single calcium sensor cannot recover from perturbations that change the periodic bursting pattern into a single spike bursting mode or even tonic spiking. One advantage of using a single sensor is that the model does not diverge as in Fig. 2 (11), but in this case, recovery from some perturbations is not possible. For this reason, we modify the model in ref. 7 so that it becomes possible to add multiple sensors without the problem of run-away activity.

### The Modified Model.

Because we know that maximal conductances must remain bounded in biological neurons, we searched for a simple modification that guarantees this physical constraint. We first considered a modification to Eq. **2** that directly addresses this:

[3] $$\begin{array}{c} \tau_{G}\frac{d\bar{g_{i}}}{\mathrm{dt}}=\left(\sum_{k}^{3}L_{\mathrm{ki}}\delta_{k}\right)\bar{g_{i}}-\gamma {\bar{g_{i}}}^{3}. \end{array}$$

The cubic term ensures that if $\bar{g_{i}}$ grows too much, then ${\bar{g_{i}}}^{3}$ becomes dominant. If $\bar{g_{i}}$ is sufficiently large, the corresponding component of the vector field [**3**] becomes negative so that the i-*th* conductance decreases. With this modification, all the conductances remain bounded to a region of conductance space *G*. The coefficient *γ* determines the tradeoff between the linear and cubic terms: If *γ* is small, conductances can grow more than with a larger value of *γ*. There is a range of values for *γ* that allow bursting solutions, and throughout this work, we kept *γ* fixed at *γ* = 10^−5^.

In the original formulation of the model (*γ* = 0), there is no true convergence (as per Fig. 2), but there is an intuition that if the sensors are close to their targets, then ∥⟨***δ***⟩∥ ≈ 0 implies that the rates of change of conductances are small, $\|\langle \overset{¯}{g}\rangle \|\approx 0$. If *γ* > 0, this is no longer the case, and therefore, there is no reason to expect that the conductances will converge to some value and remain there. To address this, we introduced a gating variable *α* that modulates the timescale of the regulation mechanism. Our approach was to combine the information of all sensors into a single feedback signal that can be used to gate the homeostatic regulation on or off. One way to achieve this is as follows:

[4] $$\begin{array}{cc} \tau_{G}\frac{d\bar{g_{i}}}{\mathrm{dt}} & =\left\{\left(\sum_{k}^{3}L_{\mathrm{ki}}\delta_{k}\right)\bar{g_{i}}-\gamma {\bar{g_{i}}}^{3}\right\}\alpha \\ \tau_{\alpha }\dot{\alpha } & =\alpha_{\infty }\left(S_{f}\right)-\alpha , \end{array}$$

where *τ*_*α*_ is the timescale of process *α* and *α*_∞_ is a sigmoid function,

[5] $$\begin{array}{c} \alpha_{\infty }\left(S_{f}\right)=\frac{1}{1+e^{-\frac{\left(-S_{f}+\rho \right)}{\Delta_{\alpha }}}}. \end{array}$$

Notice that *α* ∈ (0, 1) defines an effective timescale $\tau_{\mathrm{eff}}=\frac{\tau_{G}}{\alpha }$ such that if *α* → 0, then *τ*_*eff*_ → ∞, effectively turning off the homeostatic regulation. The parameters *ρ* and *Δ*_*α*_ correspond to the half activation and steepness of the activation function for *α*. These parameters were kept fixed at *ρ* = 0.075 *Δ*_*α*_ = 0.01 and *τ*_*α*_ = 1 s. These choices are such that values of *S*_*f*_ > 0.1 cause *α* → 0. To obtain the feedback signal dynamically, we compute the average activity of the sensors as follows:

[6] $$\begin{array}{cc} \tau_{S}\dot{E_{F}} & =\left(\overset{¯}{F}-F\right)-E_{F}\\ \tau_{S}\dot{E_{S}} & =\left(\overset{¯}{S}-S\right)-E_{S}\\ \tau_{S}\dot{E_{D}} & =\left(\overset{¯}{D}-D\right)-E_{D}. \end{array}$$

The variables *E*_*i*_ correspond to the time-averaged error or distance between the sensors’ activities to their targets: When the sensors are on-target, these quantities approach *E*_*i*_ ≈ 0. The parameter *τ*_*S*_ is the timescale over which the errors are averaged and was set to *τ*_*S*_ = *τ*_*G*_ since this is a natural choice. Our aim was to construct a single feedback signal that can be used to detect whether the activity is within target or not. This was done by combining the average error signals as follows:

[7] $$\begin{array}{c} S_{f}\left(t\right)=e^{\frac{-E_{F}^{2}}{\Delta }}\times e^{\frac{-E_{S}^{2}}{\Delta }}\times e^{\frac{-E_{D}^{2}}{\Delta }}. \end{array}$$

Here, each term corresponds to a Gaussian function with spread *Δ* = 0.001. If a sensor is close to its target, its error signal approaches 0 and the Gaussian takes values close to 1. Because we are taking a product, *S*_*f*_ can be *S*_*f*_ > 0 only in the case that the three sensors simultaneously are close to their targets. This signal therefore provides a combined measure of how close the three sensors are to their targets. The product of the Gaussians acts like an AND gate: If at least one of the sensors is off target, then *S*_*f*_ → 0.

**Fig. 4.**
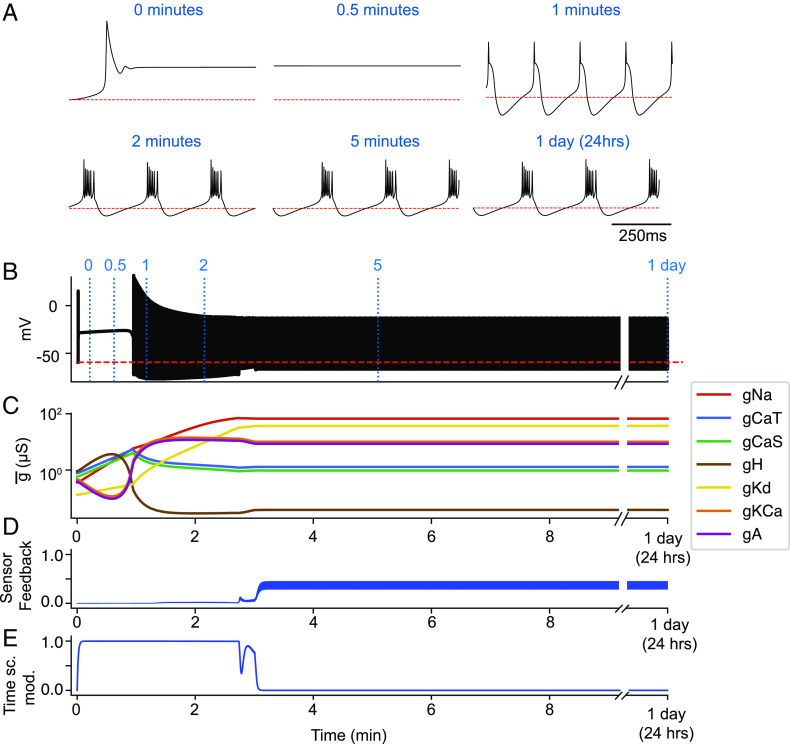
The modified model. The modified model self-assembles into a periodic bursting pattern, and once it reaches it, the homeostatic mechanism turns off. The conductances and the activity remain constant for over 24 h (≈4 × 10^4^*τ*_*G*_). (*A*) Membrane potential at different times in the simulation. (*B*) Membrane potential for the full simulation. (*C*) Conductances over time in the logarithmic scale. (*D*) Sensors feedback *S*_*f*_ signal over time. (*E*) Timescale modulation/gating variable *α* over time.

These modifications solve most of the issues with the original formulation of the original model [**2**], while preserving its valuable properties. Fig. 4 shows how the modified model works. As before, the model is started from small random initial conductances. Because initially the activity is not periodic bursting, the sensors are off-target and the homeostatic mechanism gets activated as *α* → 1, as shown in Fig. 4*E*. After a transient of about 3 min, the conductances reach values such that the activity is periodic bursting. The sensors are now reasonably close to their targets and therefore *S*_*f*_ > 0 (Fig. 4*D*), so the homeostatic mechanism is turned off (*α* → 0), and the model remains in that state for 24 h (≈4 × 10^4^*τ*_*G*_, with *τ*_*G*_ = 2 s).

### Multiple Runs and Convergence.

Both the original model [**2**] and the modified model [**4**] correspond to high-dimensional nonlinear differential equations, and a rigorous mathematical analysis of their properties is beyond the scope of this work. The intuition is that the modified model will behave in a similar way as the original model if the conductances are not too large and that once it hits a region in G space that results in periodic bursting, it will stop modifying its conductances. To test for this, we started 180 simulations from small random initial values of the conductances and let them run for 2 h (3600*τ*_*G*_, with *τ*_*G*_ = 2 s). The initial conditions for all other variables were kept fixed.

In ≈94% of the cases, the model behaved in a similar way as in Fig. 4, settling into a periodic bursting pattern after some transient behavior. The variable that determines the effective timescale of conductance evolution is *α*. In *SI Appendix*, Fig. S1 *A*, i, we show a typical (94% of cases) time series for the timescale modulation *α*(*t*). There is an initial transient where *α* > 0, and after that, *α* → 0, effectively turning off the homeostatic mechanism, because the effective timescale is now $\tau_{\mathrm{eff}}=\frac{\tau_{G}}{\alpha }$ and *τ*_*eff*_ → ∞ as *α* → 0. In a small number of cases (≈2%), we observed a temporary blip in the gating variable *α*. This is shown in *SI Appendix*, Fig. S1 *A*, ii. The cell misses a burst, and the gating mechanism turns on for a very brief period of time.

We also found that in ≈2% of the cases, the gating variable *α* does not immediately settle into equilibrium, but instead oscillates for a long time. These oscillations are shown in *SI Appendix*, Fig. S1 *A*, iii and iv. The oscillations are not periodic, and in some cases, they decay after a long transient *SI Appendix*, Fig. S1 *A*, iii. For some simulations (again ≈2% of the cases), the transient oscillation persisted beyond 2 h. The expectation however is that the oscillation will eventually stop, as it was the case for most initial conditions. *SI Appendix*, Fig. S1*B* shows these oscillations in detail: The membrane potential (*Top*) alternates between bursting and high frequency tonic spiking. This pattern activates the sensors transiently and results in an oscillation in *α* and *S*_*f*_ (*Center* and *Bottom* panels) that is not sufficient to turn off the homeostatic mechanism. In summary, the models settle into a periodic bursting pattern well before 2 h (or 3600*τ*_*G*_) in ≈98% of the instances.

*SI Appendix*, Fig. S1*C* shows the timeseries of *α* for all runs, color-coded with *α* → 0 in white and *α* > 0 in black. The panel at the *Bottom* shows the average value of *α* across initial conditions over time. Most models achieve low values of *α* in 10 min (≈300*τ*_*G*_). There are multiple periodic bursting patterns, and they are not the same. Some activate the sensors more than others. Different initial conditions land on different bursting patterns. *SI Appendix*, Fig. S1*D* shows the value of the sensor feedback signal *S*_*f*_ versus the “effective timescale” *τ*_*eff*_, or $\frac{1}{\alpha }$. The higher *S*_*f*_, the slower the effective timescale, which can become as large as 10^30^*τ*_*G*_.

### Response to Perturbations.

Perturbations are typically modeled by changing a parameter that would be considered fixed otherwise. Example perturbations include changing the reversal potential of the currents, mimicking channel deletions or applying a current blocker by setting a conductance to zero, and the injection of external current. Some perturbations disrupt the activity, while others do not appreciably affect it. A necessary condition for a model to recover from a perturbation is that this is possible, as, for example, bursting solutions (with slow waves and spikes) may not exist after blocking *Na* channels. A solution with similar activity must exist in conductance space *G* when subject to the given perturbation *G*_*P*_. Upon perturbation, the regions in *G* where bursting occurs will not in general be the same as in *G*_*P*_. If there are regions in *G*_*P*_ where the activity is recovered, then a second condition for recovery is that the homeostatic mechanism is able to successfully stabilize these regions.

To explore the model’s responses to perturbations, we focused on the periodic bursting regime as before. The perturbations we studied were 1) removing a current by setting $\bar{g_{i}}=0$, 2) changing the reversal potential of potassium *E*_*K*_, 3) changing the value of the leak conductance (*g*_*Leak*_), which is the only nonregulated conductance in this model, and 4) injecting current. We generated 12 different bursters by starting the model from random initial conditions and subjected all of them to perturbations. The models were run for 20 min with *τ*_*G*_ = 2 s. In summary, we found that the models recovered from complete removal of the *A*, *H*, *CaS*, and *CaT* currents, but they did not recover a bursting pattern if the *KCa*, *Kd*, or *Na* currents were removed. While it is possible that bursting solutions can exist without any one of these currents, the homeostatic mechanism did not find them. There is a range of values of *E*_*K*_ (−80 mV to −57 mV) for which the models can recover bursting, and similarly, there is a range of values of *g*_*leak*_ (0.01 to 0.1 nS) for which the model can also recover into a periodic bursting pattern.

**Fig. 5.**
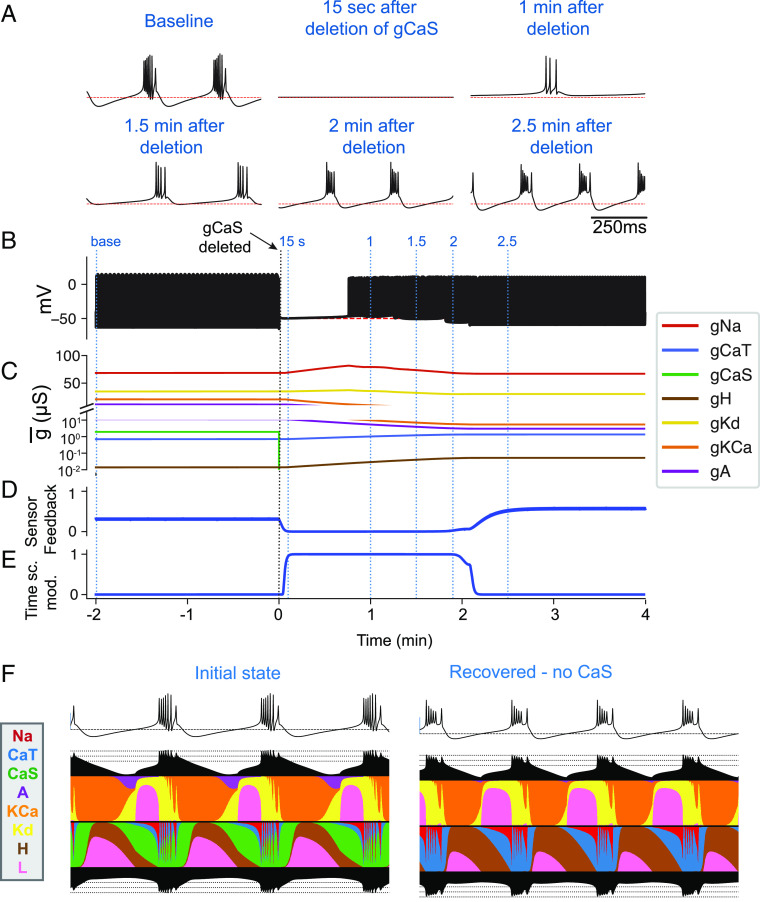
Recovery from current deletion. The model was started in a periodic bursting regime (baseline), and at time *t* = 0, the slow calcium *CaS* was removed. The model initially becomes hyperpolarized and quiescent, and therefore, the homeostatic mechanisms turn on *α* → 1. The model recovers its target bursting pattern by minute 2.5 and the homeostatic mechanism turns off (*α* → 0). (*A*) Membrane potential at different points in time. (*B*) Membrane potential for the full simulation. (*C*) Maximal conductances over time. The y-axis is broken. We used a linear scale (*Top*) for the range 25 nS to 100 nS and a logarithmic scale (*Bottom*) for the range 10^−2^*nS* to 25 nS. (*D*) Sensor feedback *S*_*f*_ over time. (*E*) Timescale modulation *α* over time. (*F*) Currentscapes in the control (*Left*) and adapted (*Right*) conditions. The currentscapes show the percent contribution of each current type to the activity over time (22).

Fig. 5 illustrates how the model can recover from perturbations. In this example, we removed the slow calcium current *CaS*. The model was started at a periodic bursting regime, and at time *t* = 0, we set *g*_*CaS*_ = 0 for the rest of the simulation. Initially, the model became quiescent (15 s after deletion). Because the activity was not on target, *S*_*f*_ → 0 (Fig. 5*D*), and therefore, the homeostatic mechanism turned on *α* → 1 (Fig. 5*E*). As a consequence, the maximal conductances changed over time (Fig. 5*C*), and about 1 min after deletion (30*τ*_*G*_), the model regained spiking activity. This spiking activity was not sufficient to fully activate the sensors; therefore, *S*_*f*_ ≈ 0, and the conductances continued to evolve. At minute 2.5 (about 75*τ*_*G*_) after deletion, the model achieved its target periodic bursting activity, the sensor feedback was high *S*_*f*_ > 0.1, the homeostatic mechanism turned off (*α* → 0), and the conductances remained constant for the rest of the simulation. The *Bottom* panels (*F*) show currentscapes for the control (*Left*) and adapted (*Right*) states. The currentscapes show the percent contribution of each current type (in colors) to the activity over time (22). Notice that initially there is a visible contribution of the *A* current (in purple), there is a large contribution of the *CaS* current (in green), and a small contribution of the *CaT* current (in blue). Upon removal of *CaS*, the homeostatic mechanism changes the maximal conductances, and all currents get rearranged to produce the target bursting pattern. In the adapted state (*Right*), the contribution of the *A* current is negligible, and the slow calcium current *CaS*, which is no longer present, was replaced by a large contribution of the other calcium current *CaT*.

### Different Types of Perturbations Elicit Similar Changes in Membrane Potential but Trigger Different Internal Responses to Recover and Adapt.

In a similar way as its original version (7), the modified model [**4**] can recover from multiple different types of perturbations. However, recovering from different perturbations may require different strategies. Perhaps less intuitive is that perturbations that elicit similar changes in membrane potential activity trigger different changes in conductance densities. To show this, we subjected the models to two different depolarizing perturbations and let them recover.

**Fig. 6.**
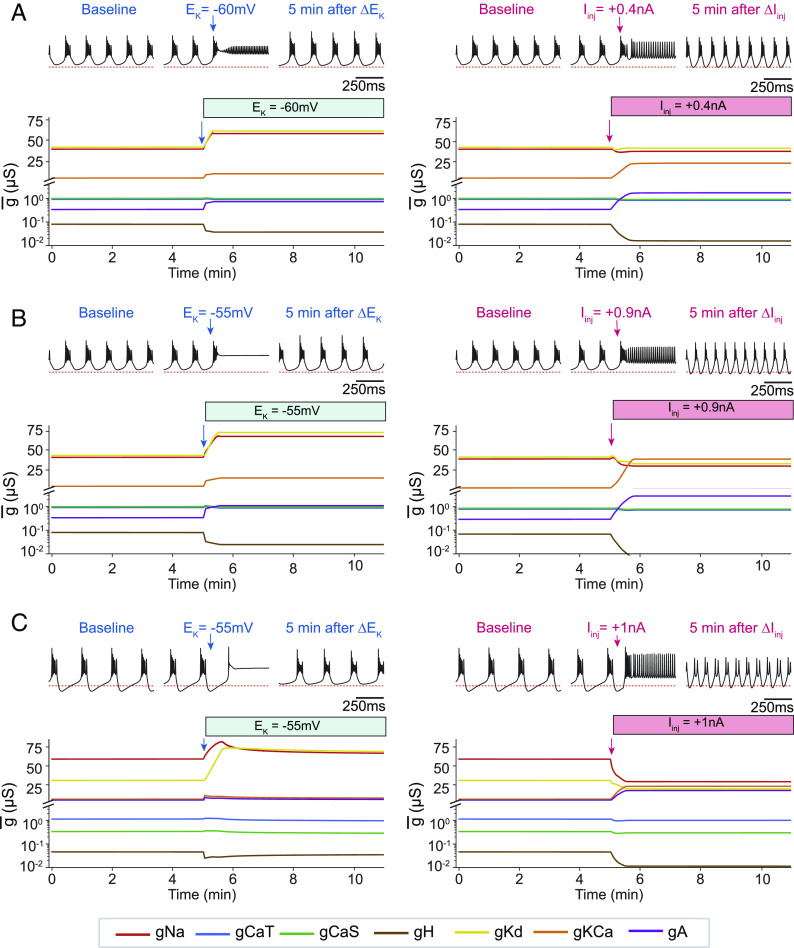
Different types of perturbations elicit similar changes in membrane potential but trigger different internal responses to recover and adapt. The model was started in a periodic bursting regime and subject to depolarizing perturbations. The figure compares the responses of the model to changes in the reversal potential of potassium *E*_*K*_ (*Left*) and depolarizing current injections *I*_*app*_ (*Right*). *A* (*Top*) membrane potential at different points in time (*Left*: control; *Center*: initial response; *Left*: adapted/recovered). (*Bottom*) Conductances over time. *B* Idem (*B*) but increasing the strength of both perturbations (see main text). The initial state of the model is identical to that in (*A*). *C* Idem (*A*) but for a model started from different initial conductances.

In Fig. 6*A*, we show the response of the model to changing the reversal potential of potassium *E*_*K*_ from *E*_*K*_ = −80 mV in control, to *E*_*K*_ = −60 mV, and compare it to its response to a depolarizing current injection *I*_*app*_ = 0.4 nA. The instantaneous response of the model is similar in both cases: The membrane depolarizes, and the cell fires tonically. As before, this triggers changes in the conductance densities in an attempt to recover bursting activity. In both cases, the model is able to adapt to the perturbed environment, but it does so by different mechanisms. In the case of *E*_*K*_, the *Na* and *Kd* conductances were increased and the *KCa* conductance changed very little, while in the case of *I*_*app*_, the *Na* and *Kd* conductances remained almost constant and the *KCa* conductance increased notably.

The extent by which these changes differ depends on the extent of the perturbation. In Fig. 6*B*, we show the same model as in Fig. 6*A*, but the strength of the perturbations was increased. In Fig. 6*B*, we changed *E*_*K*_ from *E*_*K*_ = −80 mV in control to *E*_*K*_ = −55 mV and *I*_*app*_ = 0 in control to *I*_*app*_ = 0.9 nA. The response of the model was qualitatively similar as in Fig. 6*A*, but the changes in conductance densities were larger.

Both the instantaneous and long-term responses of the model to a perturbation depend on the values of the maximal conductances that determine its excitability. In Fig. 6*C*, we considered a model whose initial conductances are different from those in Fig. 6 *A* and *B* and subjected it to the same perturbation paradigms. Even though the perturbation depolarizes the cell in both cases, the instantaneous responses differ in that this model became quiescent for the *E*_*K*_ case, while it spiked tonically for the *I*_*app*_ case. Despite this, the changes in conductances are qualitatively similar to the previous model (Fig. 6 *A* and *B*). The *Na* and *Kd* conductances increased, and *KCa* changed little in the case of the *E*_*K*_ perturbation, while the opposite happened for the *I*_*app*_ perturbation.

Naturally, it is unfeasible to perform an exhaustive exploration of the responses to perturbations for all possible bursting solutions that the current model can attain. However, these examples illustrate that the homeostatic response of the model to a perturbation is in general different across perturbation types, even in the case that the changes in membrane potential are similar, and that this can also be the case in models for which the instantaneous response to perturbations are different (30).

### Long-Term Storage of Prior Perturbations.

In general, for a model to adapt and function during a perturbation, its conductances—and therefore its excitability—must change. When the perturbation is removed, the conductances of the model will be different from its starting or control conductances, and its activity may or may not be the target periodic bursting regime that will activate the sensors. Therefore, when the perturbation is removed, the model must somehow return to a bursting regime. Because there are multiple values of the conductances that produce any desired periodic bursting activity, the model is likely to return to a different point in conductance space and therefore accrue changes in its conductance densities. When a second perturbation is applied, the model’s instantaneous response will in general be different because its conductances are different.

**Fig. 7.**
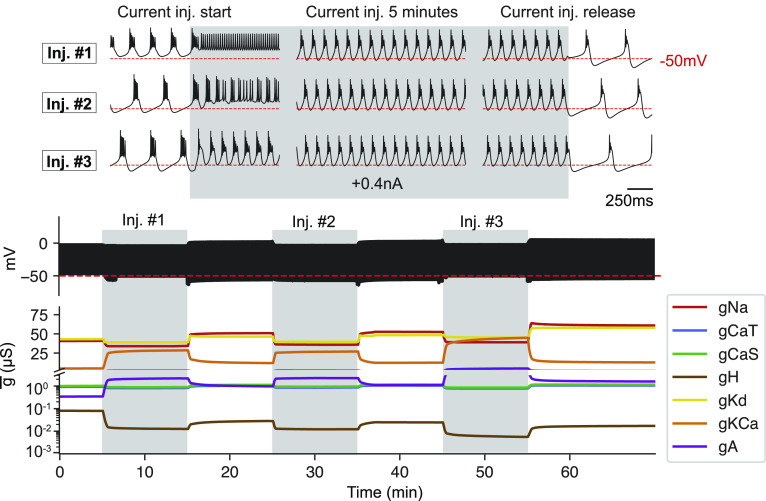
Multiple applications and accrual. We subjected the model to three identical depolarizing current injections *I*_*app*_. The initial response of the model to the perturbation is history dependent. (*Top*) Membrane potential at different points in time. (*Bottom*) Maximal conductances over time (solid lines) and reference values (dashed lines).

To illustrate this, in Fig. 7, we subjected the model to the same depolarizing current injection *I*_*app*_ three times. The initial response of the model to the depolarizing current injection is to switch from periodic bursting to tonic firing. Because the sensors are off-target, the homeostatic mechanism turns on (*α* → 1), and the conductances change in such a way that the model regains its periodic bursting activity during the current injection. When the perturbation is removed, the model bursts periodically, but the slow wave is more hyperpolarized than in control. The sensors are not satisfied by this particular waveform, and this turns on the homeostatic mechanism in an attempt to bring the model closer to its control activity. Over time, the model settles in a periodic bursting regime that satisfies the sensors enough that the mechanism is turned off (*α* → 0), but the maximal conductances at which the model stabilizes are different from those in control. Note also that while qualitatively the model is doing the same as in control, the waveform is slightly different. When the second perturbation is applied, the instantaneous response of the model is not tonic spiking, but instead, it displays irregular bursts. As before, the sensors are not satisfied by this activity, and this triggers changes in the conductances. Because the starting conductances at the time of the second perturbation are different from those in control (first perturbation), the homeostatic mechanism produces a slightly different response, and the model adapts during the perturbation with slightly different values of its maximal conductances. By the same token, when the perturbation is removed, the changes in the maximal conductances needed to return close to control activity are also different from those in the first release. The instantaneous response of the model to a third application is again different from the previous two applications, and it is able to retain a periodic bursting pattern upon current injection. This instantaneous bursting pattern does not fully satisfy the sensors, and thus, the model changes its conductances again to burst periodically during the perturbation. This example shows that while the model can recover and adapt to a number of perturbations, its excitability properties when the perturbation is removed can be different from those in control. These changes in excitability are revealed by the instantaneous response to subsequent perturbations.

## Discussion

Understanding how neurons regulate their intrinsic excitability is fundamental to understanding the role each neuron plays in the circuits in which it is found. The first models of homeostatic regulation of intrinsic excitability were important because they articulated the principle that ion channel densities had to be regulated in a coordinated fashion, as there are degenerate sets of conductances that can provide similar, if not identical, neuronal outputs (4, 6, 7, 10, 11, 22, 24, 31).

In this paper, we reformulate the Liu et al. (7) model to address directly some of the interesting constraints provided by biological neurons. The original model is susceptible to run-away losses of stability such that its conductances increase exponentially way beyond levels of channel density that could conceivably be found in biological measurements (7, 10, 11, 22, 24). Consequently, we reasoned that the properties of proteins and membranes create a hard *upper*-bound on possible conductance densities, which motivated the nonlinear degradation term we now implement on conductance densities in this model formulation.

Replacing ion channels in the membrane is energy consuming. In most previous homeostatic models of intrinsic excitability, the unspoken premise is that the turnover of the channel proteins would be primarily determined by the need to regulate the neuron’s excitability. But, there are a myriad of biological processes that require energy, and it is likely that replacement of ion channel proteins will also depend on the status of a number of other biological processes. Significantly, one of the innovations of the present work is that the homeostatic process is effectively turned off when the neuron is close to its activity targets. This would allow the neuron to deploy less energy to unnecessary channel turnover and free that energy for other demands. The implication of this is that there needs to be a threshold deviation from target activity that determines when the homeostatic mechanism is turned on/off.

We report here that different perturbations that result in similar changes in activity (Fig. 7) nonetheless produce different changes in conductances. In the example shown in this paper, we show that depolarization with current injection, and depolarization with high *K*^+^ concentrations, do not produce equivalent changes in conductance densities. This argues that extreme caution needs to be used when using one perturbation as a proxy for another. While we show this here for high *K*^+^ and current injection, we expect that the same principle will hold when comparing the results of synaptic inputs and current injection. This caution will be important for all those using optogenetic and other light activation of neurons as a proxy for the activation of the same neurons by their natural synaptic inputs.

Another important feature of this model is that it shows traces of prior perturbations that may be “cryptic” or not obviously visible in the activity patterns under control or baseline conditions but may be revealed by subsequent perturbations. This occurs because recovery from perturbation need not return the model’s conductances to their starting sets of values but are far more likely to find another, also successful, solution. This helps explain the pronounced accrual seen in recent experiments with multiple high *K*^+^ applications (32), even when interleaved by substantial washes with control saline. If the spirit of this model captures the general features shown by biological systems, once a neuron has recovered its control activity, if the homeostatic process is effectively turned off, this will prolong the time over which the neuron preserves its recovered set parameters, thus creating a “memory” of prior perturbations that may not be visible until the neuron is once again perturbed (32). The accrual of cryptic changes that may only be revealed by subsequent perturbations is potentially significant for both understanding the etiology of some posttraumatic stress disorders or the long-term changes in excitability and network function in response to climate change (33).

matseccnt1

## Methods

## Materials and Methods

The Liu et al. (7) models’ dynamics is given by a Hodgkin–Huxley equation for a single compartment with eight currents. The neuron has a sodium current, *I*_*Na*_; transient and slower calcium currents, *I*_*CaT*_ and *I*_*CaS*_, respectively; a transient potassium current, *I*_*A*_; a calcium-dependent potassium current, *I*_*KCa*_; a delayed rectifier potassium current, *I*_*Kd*_; a hyperpolarization-activated inward current, *I*_*H*_; and a leak current, *I*_*leak*_. The membrane potential *V* of a cell containing *N* channels and membrane capacitance *C* is given by

[8] $$\begin{array}{c} C\frac{\mathrm{dV}}{\mathrm{dt}}=I_{e}-\sum_{i=1}^{8}I_{i}. \end{array}$$

Each term in the sum corresponds to an ionic current $I_{i}=\bar{g_{i}}m_{i}^{p_{i}}h_{i}^{q_{i}}\left(V-E_{i}\right)$, and *I*_*e*_ is externally applied current. The maximal conductance of each channel is given by $\bar{g_{i}}$. *m*_*i*_, and *h*_*i*_ are the activation and inactivation variables, the integers *p*_*i*_ and *q*_*i*_ are the number of gates in each channel, and *E*_*i*_ is the reversal potential of the ion associated with the i-*th* current. The reversal potentials of the Na, K, H, and leak currents were *E*_*Na*_ = 30 mV, *E*_*K*_ = −80 mV, *E*_*H*_ = −20 mV, and *E*_*leak*_ = −50 mV, while the calcium reversal potential *E*_*Ca*_ was computed dynamically using the Nernst equation assuming an extracellular calcium concentration of 3mM. The kinetic equations describing the seven voltage-gated conductances were modeled as in ref. 7:

[9] $$\begin{array}{cc} \tau_{m_{i}}\left(V\right)\frac{dm_{i}}{\mathrm{dt}} & =m_{\infty_{i}}\left(V\right)-m_{i}\\ \tau_{h_{i}}\left(V\right)\frac{dh_{i}}{\mathrm{dt}} & =h_{\infty_{i}}\left(V\right)-h_{i}. \end{array}$$

The functions *τ*_*m*_*i*__(*V*), *m*_∞_*i*__(*V*), *τ*_*h*_*i*__(*V*), and *h*_∞_*i*__(*V*) are based on experimental work (34). The activation functions of the *K*_*Ca*_ current require a measure of the internal calcium concentration [*Ca*^+2^] (7). The dynamics of the intracellular calcium concentration are given by

[10] $$\begin{array}{c} \tau_{\mathrm{Ca}}\frac{d[Ca^{+2}]}{\mathrm{dt}}=-Ca_{F}\left(I_{\mathrm{CaT}}+I_{\mathrm{CaS}}\right)-\left[Ca^{+2}\right]+Ca_{0}. \end{array}$$

Here, *Ca*_*F*_ = 0.94 $\frac{\mu M}{\mathrm{nA}}$ is a current-to-concentration factor and *Ca*_0_ = 0.05 *μM*. Finally, the total capacitance of the cell is *C* = 1*nF*.

In this model, the conductances are state variables and evolve according to Eq. **2** in Liu et al. (7),

$$\begin{array}{c} \tau_{G}\frac{d\bar{g_{i}}}{\mathrm{dt}}=\left[A_{i}\left(\overset{¯}{F}-F\right)+B_{i}\left(\overset{¯}{S}-S\right)+C_{i}\left(\overset{¯}{D}-D\right)\right]g_{i}. \end{array}$$

The sensors’ target values were kept fixed throughout this work at $\left[\overset{¯}{F},\overset{¯}{S},\overset{¯}{D}]=[0.1,0.07,0.07\right]$. The activities of the sensors are given by the following expressions:

[11] $$\begin{array}{cc} F & =G_{F}M_{F}^{2}H_{F}\\ S & =G_{S}M_{S}^{2}H_{S}\\ D & =G_{D}M_{D}^{2}, \end{array}$$

where the *M*_*X*_ and *H*_*X*_ (with *X* = *F*, *S*, or *D*) variables represent activation and inactivation, respectively. We set *G*_*F*_ = 10, *G*_*S*_ = 3, and *G*_*D*_ = 1 (7). The activation and inactivation variables *M*_*X*_ and *H*_*X*_ follow

[12] $$\begin{array}{cc} \tau_{M_{X}}\frac{dM_{X}}{\mathrm{dt}} & =M_{X}\left(I_{\mathrm{Ca}}\right)-M_{X}\\ \tau_{H_{X}}\frac{dH_{X}}{\mathrm{dt}} & =H_{X}\left(I_{\mathrm{Ca}}\right)-H_{X}. \end{array}$$

The timescales *τ*_*M*_ and *τ*_*H*_ control the frequency range over which a sensor is sensitive to changes in the calcium current *I*_*Ca*_ = *I*_*CaT*_ + *I*_*CaS*_. In this work, we used values identical to those in ref. 7, which we reproduced in Table 2.

**Table 2.** Values of *Z* parameters used in ref. 7

Parameter	*Z*_*M*_	*Z*_*H*_	*τ*_*M*_	*τ*_*H*_
F	14.8	9.8	0.5 ms	1.5 ms
D	7.2	2.8	50 ms	60 ms
S	3	–	500 ms	–

The functions *M*_*X*_(*I*_*Ca*_) and *H*_*X*_(*I*_*Ca*_) are given by

[13] $$\begin{array}{cc} M_{X}\left(I_{\mathrm{Ca}}\right) & =\frac{1}{1+e^{Z_{M_{X}}+\frac{I_{\mathrm{Ca}}}{1nA/nF}}}\\ H_{X}\left(I_{\mathrm{Ca}}\right) & =\frac{1}{1+e^{-Z_{H_{X}}-\frac{I_{\mathrm{Ca}}}{1nA/nF}}}. \end{array}$$

All parameters and equations were identical to Liu et al. (7), except for the modifications described in *Results*. The conductances in the modified model evolve according to Eq. **4**

$$\begin{array}{cc} \tau_{G}\frac{d\bar{g_{i}}}{\mathrm{dt}} & =\{\left(\sum_{k}^{3}L_{\mathrm{ki}}\delta_{k}\right)\bar{g_{i}}-\gamma {\bar{g_{i}}}^{3}\}\alpha \\ \tau_{\alpha }\dot{\alpha } & =\alpha_{\infty }\left(S_{f}\right)-\alpha , \end{array}$$

with

$$\begin{array}{c} \alpha_{\infty }\left(S_{f}\right)=\frac{1}{1+e^{-\frac{\left(-S_{f}+\rho \right)}{\Delta_{\alpha }}}}, \end{array}$$

and

$$\begin{array}{cc} \tau_{S}\dot{E_{F}} & =\left(\overset{¯}{F}-F\right)-E_{F}\\ \tau_{S}\dot{E_{S}} & =\left(\overset{¯}{S}-S\right)-E_{S}\\ \tau_{S}\dot{E_{D}} & =\left(\overset{¯}{D}-D\right)-E_{D}, \end{array}$$

and

$$\begin{array}{c} S_{f}\left(t\right)=e^{\frac{-E_{F}^{2}}{\Delta }}\times e^{\frac{-E_{S}^{2}}{\Delta }}\times e^{\frac{-E_{D}^{2}}{\Delta }}. \end{array}$$

In all figures, the model was integrated using an exponential Euler scheme (35) with step *dt* = 0.1 ms. The model and the integration routine were implemented in Python. The solutions were visualized and analyzed using standard Python libraries (numpy and matplotlib).

## Supplementary Material

Appendix 01 (PDF)

## Data Availability

Code is available at https://github.com/leandro-alonso/homeostasis.
