# Supplementary material for: Gating of homeostatic regulation of intrinsic excitability produces cryptic long-term storage of prior perturbations
Source: Proc Natl Acad Sci U S A. 2023 Jun 20;120(26):e2222016120. doi: 10.1073/pnas.2222016120 (PMC10293857; doi:10.1073/pnas.2222016120)
Supplement: Supplementary file 1 — Appendix 01 (PDF) [file pnas.2222016120.sapp.pdf]

Supporting Information for

Gating of homeostatic regulation of intrinsic excitability produces cryptic long-term storage of prior perturbations

Leandro M. Alonso, Mara C.P. Rue and Eve Marder

Eve Marder.
E-mail: marder@brandeis.edu
Leandro M. Alonso.
E-mail: lalonso@brandeis.edu

This PDF file includes:

Supporting text
Fig. S1

Supporting Information Text

Subhead. Supplemental Figure 1 summarizes the behavior of the model (**??**) when started from *small random* initial conductances and integrated for 2 hours ($3600\tau_G$). Panel (A) shows the time series of $\alpha(t)$ for four cases (see main text). In most cases the model quickly converges into a periodic bursting pattern and stays there for the rest of the simulation ($\alpha \rightarrow 0$). In some cases $\alpha(t)$ displays oscillatory behavior that can last for over 2 hours. Panel (B) investigates such cases. The top panel shows the membrane potential, the center panel shows $\alpha(t)$ and the bottom panel shows the feedback signal $S_f(t)$. Panel (C, top) shows $\alpha(t)$ for all runs: here $\alpha \approx 1$ is indicated in black while $\alpha \approx 0$ is indicated in white. Panel (C, bottom) shows $\alpha(t)$ averaged across initial conditions. Most models ($\approx 94\%$) achieve the target pattern in $\approx 10 mins$ ($\approx 300\tau_G$). Finally, panel (D) shows the effective timescale $\frac{1}{\alpha}$ as a function of the sensor feedback S_f at the end of the simulation. The points close to the origin correspond to the cases in which there is persistent oscillatory activity and the model did not yet converge.

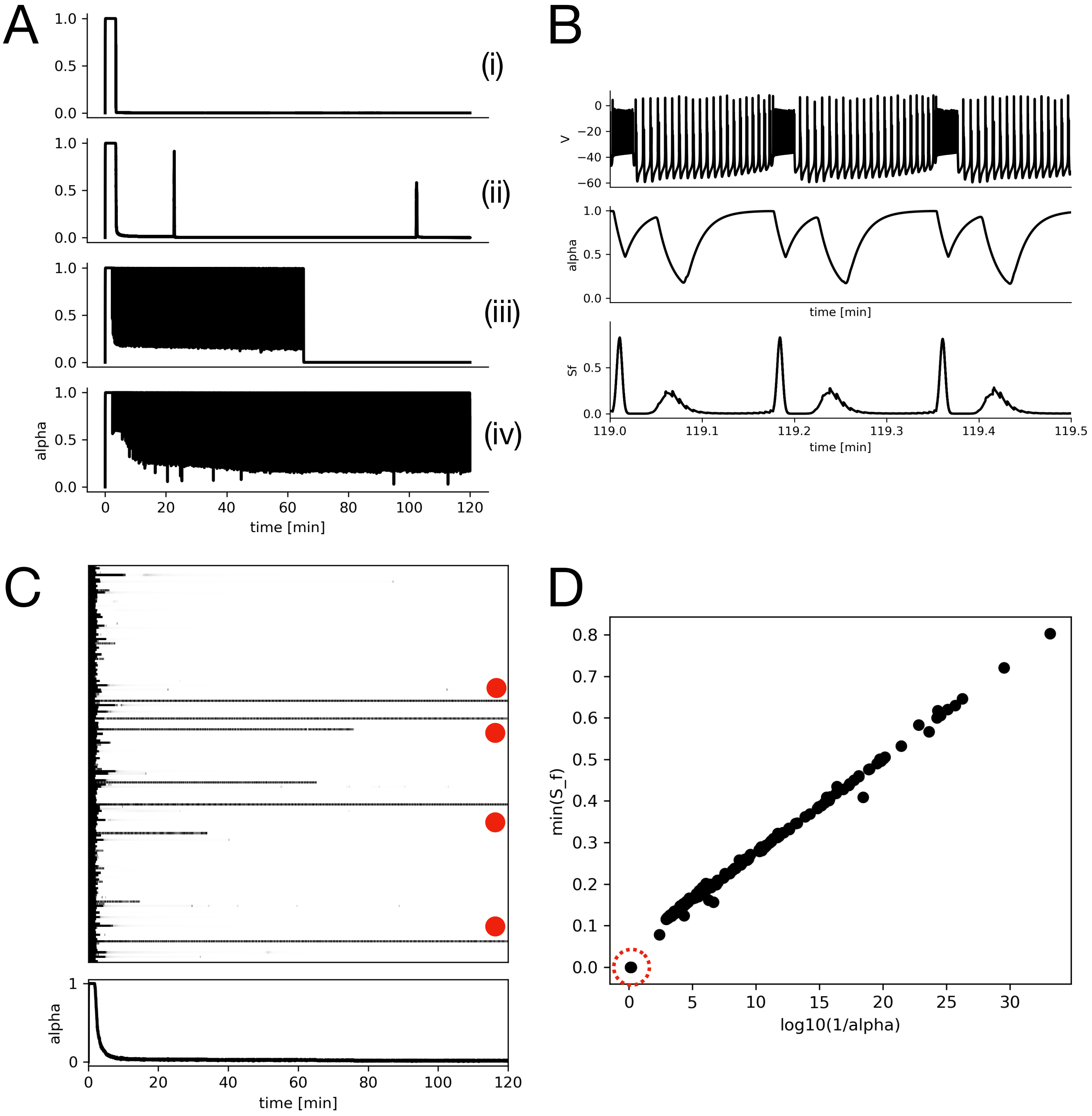

Fig. S1. Multiple runs from random small initial conductances. The model was started from *small random* initial conductances and let evolve for 2 hours ($\approx 3600\tau_G$). In most cases ($\approx 94\%$) the model self-assembles into a periodic bursting pattern after a transient of about $300\tau_G$. For some initial conditions (2%) this transient can last for longer than 2 hours. **A** Time series of $\alpha(t)$ for different behaviors (i-iv). **B** Detailed view of membrane potential **(top)**, effective timescale $\alpha(t)$ **(middle)**, and sensor feedback $S_f(t)$ during a transient oscillation **(bottom)**. **C (top)** $\alpha(t)$ for all initial conditions colorcoded ($\alpha \approx 1$ in black and $\alpha \approx 0$ in white). **(bottom)** average α across initial conditions. **D** Effective timescale $\frac{1}{\alpha}$ (in log scale) versus sensor feedback $S_f(t)$. The red dashed circle indicates the solutions that failed to stabilize in 2 hours (these are also indicated by red filled circles in C).
